# Bacillary Partnerships

**Published:** 1891-04

**Authors:** 


### Bacillary Partnerships.

In the course of some experimental investigations on the relationship of micro-organisms with diseased conditions, Drs. Cornil and Babes have discovered that a certain affinity exists between particular species. In other words, the development of special varieties may be facilitated, or the reverse, by the presence or pre-existence of certain other varieties. In this way the occasional complication of an existing infectious disease by a second is not the result of mere chance, but is governed by some still undefined conditions of environment. In other instances this association of two or more species of micro-organisms is necessary to the evolution of the malady. This association is the rule in the infectious diseases of human beings, and it is often the secondary infection that determines the fatal issue. This partnership arrangement may take place between microbes belonging to more or less nearly related species, as in the case with the organisms of pneumonia and typhoid fever. Or there may be streptococci and bacilli together, as in diphtheria, or several varieties of streptococci, as in the infection of wounds. In fact, there is a large selection of these associations, some invariable, others frequent, and a third category in which the secondary infection is accidental. These facts may possibly throw some light on the rythm and sequence of the symptoms in the infectious diseases.—*Medical Press.*

Micro-organisms of the Human Mouth. By Willoughby D. Miller, D.D.S., M.D. What the *Medical Record* says about it. Some idea of the thoroughness with which this work has been prepared is gained by a glance at the bibliography which contains about two hundred and fifty references. Part I of the volume comprising general bacteriolgical studies, with special reference to the bacteria of the human mouth. A brief but comprehensive discussion is given of the forms of bacteria, their origin, reproduction, life conditions, and their behavior toward various chemical and physical reagents. Their vital manifestations are given at some length. No less than seven substances found in the mouth serve as nutrient media for their reproduction. Among these are the normal saliva and mucus, softened dental tissue, and accumulated particles of food. Full directions are given for the proper conduct of studies and experiments along the lines indicated. Chapter VII is entitled "Original Investigations on the Decay of the Teeth," and naturally attracts the most attention. Part II treats of the "Pathogenic Mouth Bacteria and the Diseases which they Produce."

We have not space to speak in detail of Dr. Miller's conclusions. They are of interest not only to dentists, but to all engaged in bacteriological work. From his name we conclude that he is an American, and we are glad to have such an able representative at one of the largest and most famous of the European universities.

---

In using chloroform as an anæsthetic the decomposition of the vapor in the presence of artificial light, especially gas, produces irritating and noxious fumes. These have been analyzed by Kunkel and found to consist principally of hydrochloric acid. Where free ventilation is not possible, water or lime water, or a solution of soda, exposed in a basin or sprayed will neutralize the acid.—*Med. and Surg. Reporter.*

---

The medical as well as the dental profession are pestered with sensational doctors. In Omaha, so it is said, a free entertainment and polyclinic is conducted by "German doctors" in one of the theatres. Admission is free, and tumors are removed or cross-eyes streightened in full view of the audience. "The entertainment is pleasing and instructive" (?), and no doubt morbid curiosity is gratified.

---

THE proceedings of the recent meeting of the Mississippi Valley Dental Society will be published in the next Number of the REGISTER.

